# Paediatric Cervical Osteomyelitis With an Atypical Presentation: A Case Report and Literature Review

**DOI:** 10.7759/cureus.100077

**Published:** 2025-12-25

**Authors:** Amal Halim Salib Hanna, Shafiya Jahan Mohamed Tajuddin, Jasmine Churms, Sunitha Peiris

**Affiliations:** 1 Paediatrics, Mid and South Essex NHS Foundation Trust, Basildon, GBR; 2 General Medicine, Blackpool Teaching Hospitals NHS Foundation Trust, Blackpool, GBR; 3 Paediatrics, Blackpool Teaching Hospitals NHS Foundation Trust, Blackpool, GBR

**Keywords:** cervical osteomyelitis, delayed presentation, neck pain, paediatric spinal infection, prolonged antibiotic therapy

## Abstract

Cervical osteomyelitis is an uncommon condition in children. Diagnosis can prove difficult due to non-specific symptoms. We describe a case of paediatric cervical osteomyelitis that initially manifested with neck spasms, later progressing to fever and lymphadenitis, illustrating the variable clinical course of the condition. Investigations showed elevated inflammatory markers. The initial blood culture was reported as a contaminant, with subsequent blood cultures remaining negative. Magnetic resonance imaging (MRI) confirmed the diagnosis of cervical osteomyelitis at the C3-C4 level. The patient required prolonged intravenous (IV) antibiotics for 16 weeks due to persistent symptoms and MRI findings. Management was guided by a Multidisciplinary Team (MDT). The patient made a full recovery without any residual complications.

This case highlights the importance of timely diagnosis and appropriate management for a favourable prognosis in children with cervical osteomyelitis.

## Introduction

Spinal infections in children, particularly vertebral osteomyelitis, are rare conditions, with the latter accounting for only about 1%-2% of cases in the paediatric population [1-3]. More specifically, cervical involvement is less common compared to the thoracic and lumbar regions [2,3]. The condition often presents with nonspecific symptoms, which results in diagnostic delay [1,3]. Treatment modalities mostly involve medical management, while surgical interventions may also be required in cases with complications such as abscess formation, spinal instability, or neurological compromise [1,4].

We present an eight-year-old diagnosed with cervical osteomyelitis without an identifiable source. This case report illustrates the challenges in diagnosing paediatric cervical osteomyelitis and highlights the complexities of treatment in children, including challenges with adherence and complications related to intravenous (IV) access. It also emphasises the critical role of a Multidisciplinary Team (MDT) in achieving optimal outcomes.

## Case presentation

A previously well eight-year-old male presented with a two-week history of gradually worsening right-sided neck pain. There was no recent travel history. He was initially diagnosed with a sport-related musculoskeletal injury and was prescribed analgesia in primary care. However, his symptoms progressed, and he began experiencing intermittent, severely painful right-sided neck spasms lasting up to 40 minutes. He was reassessed by a different General Practitioner and treated as torticollis with oral diazepam, with minimal improvement in symptoms.

**Figure 1:**
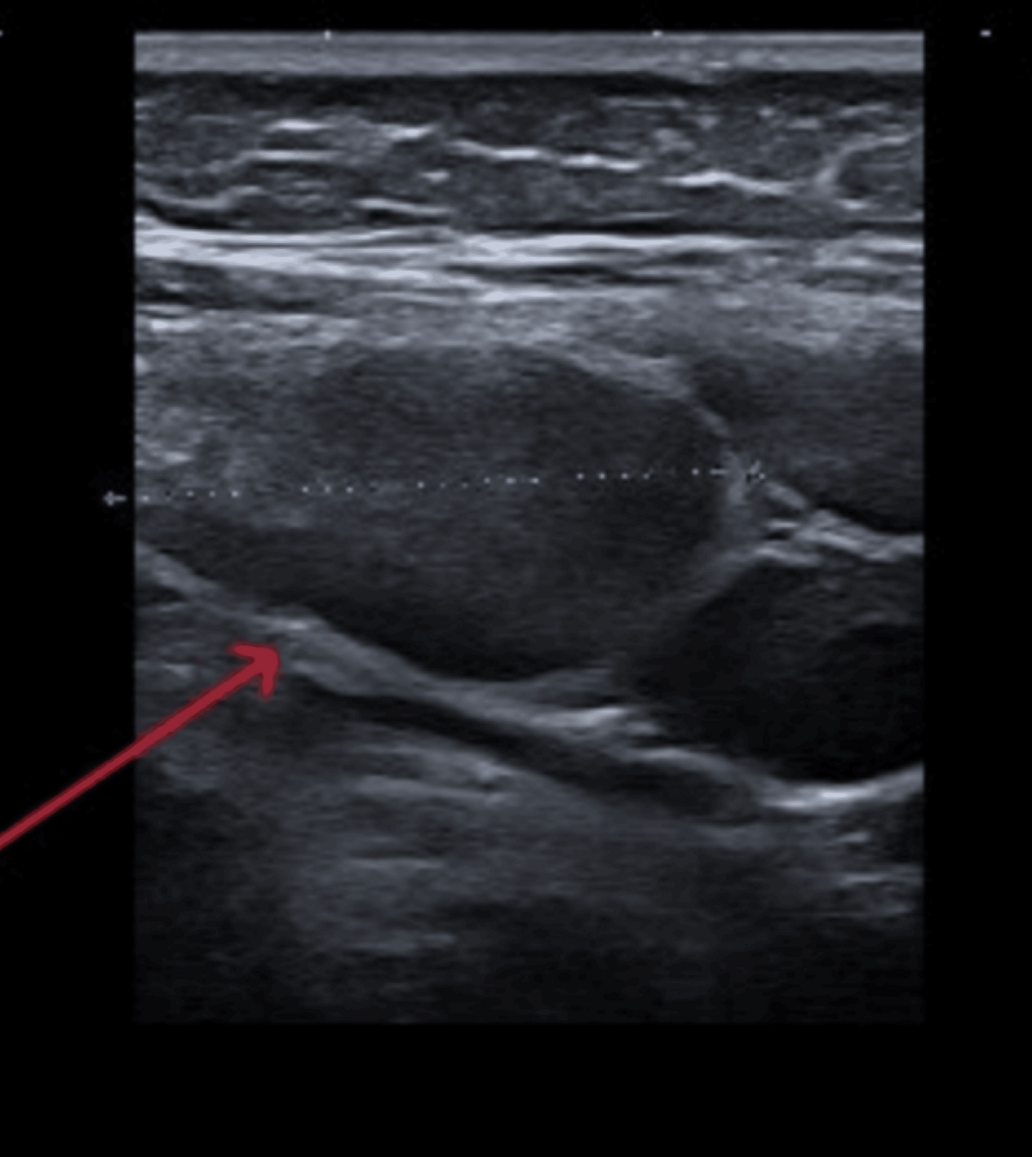
Ultrasound image of the neck showing right-sided enlarged lymph nodes (arrow)

Following two weeks of persistent symptoms, he was referred to the Paediatric Team. On initial assessment, he was afebrile, exhibited no rash or photophobia, but did demonstrate significant tenderness on palpation of his neck. He held his neck tilted to the right side but retained full passive and active neck movements. There were no signs of erythema or warmth, but enlarged cervical lymph nodes were palpable on neck examination. His neurological examination was unremarkable. Additionally, there was tenderness at the base of the right neck, particularly in the anterior aspect of the trapezius muscle, without any signs of neck stiffness. The differential diagnoses considered were torticollis versus lymphadenitis. Blood tests, including blood culture, were performed, showing an initial C-reactive protein (CRP) of 37.4 mg/L (0.2-4.9 mg/L) and white cell count (WCC) of 8.5 × 10^9^/L (4.8-12.0 × 10^9^/L). Neck ultrasound (US), done on the day of presentation, showed bilateral lymphadenopathy, likely reactive, with no abscess formation identified (Figure 1). He was started on IV ceftriaxone (50 mg/kg) and was referred to continue it in the community. The child re-presented the following day with severe, worsening neck pain and a fever of 38.4°C. He was admitted for observation, and antibiotics were continued alongside analgesia. Blood culture from the day of presentation initially came back positive for Gram-positive coagulase-negative cocci (*Staphylococcus epidermidis*), which was reported by the Microbiology Team as a likely contaminant. On day 4, following microbiology consultant advice, IV metronidazole was added to the antimicrobial regimen. A repeat blood culture obtained on day 5 of admission was negative.

A specialist opinion from the local Ear, Nose, and Throat (ENT) Team was obtained. Examination of the ears and oropharynx was unremarkable, except for palpable lymph nodes in the right posterior triangle of the neck. No obvious sinister ENT pathology was suspected, but recommendations were given for computerised tomography (CT) or magnetic resonance imaging (MRI) to exclude any deep-seated infection. The decision to perform an MRI, rather than a CT, was made in consultation with a consultant radiologist.

**Figure 2:**
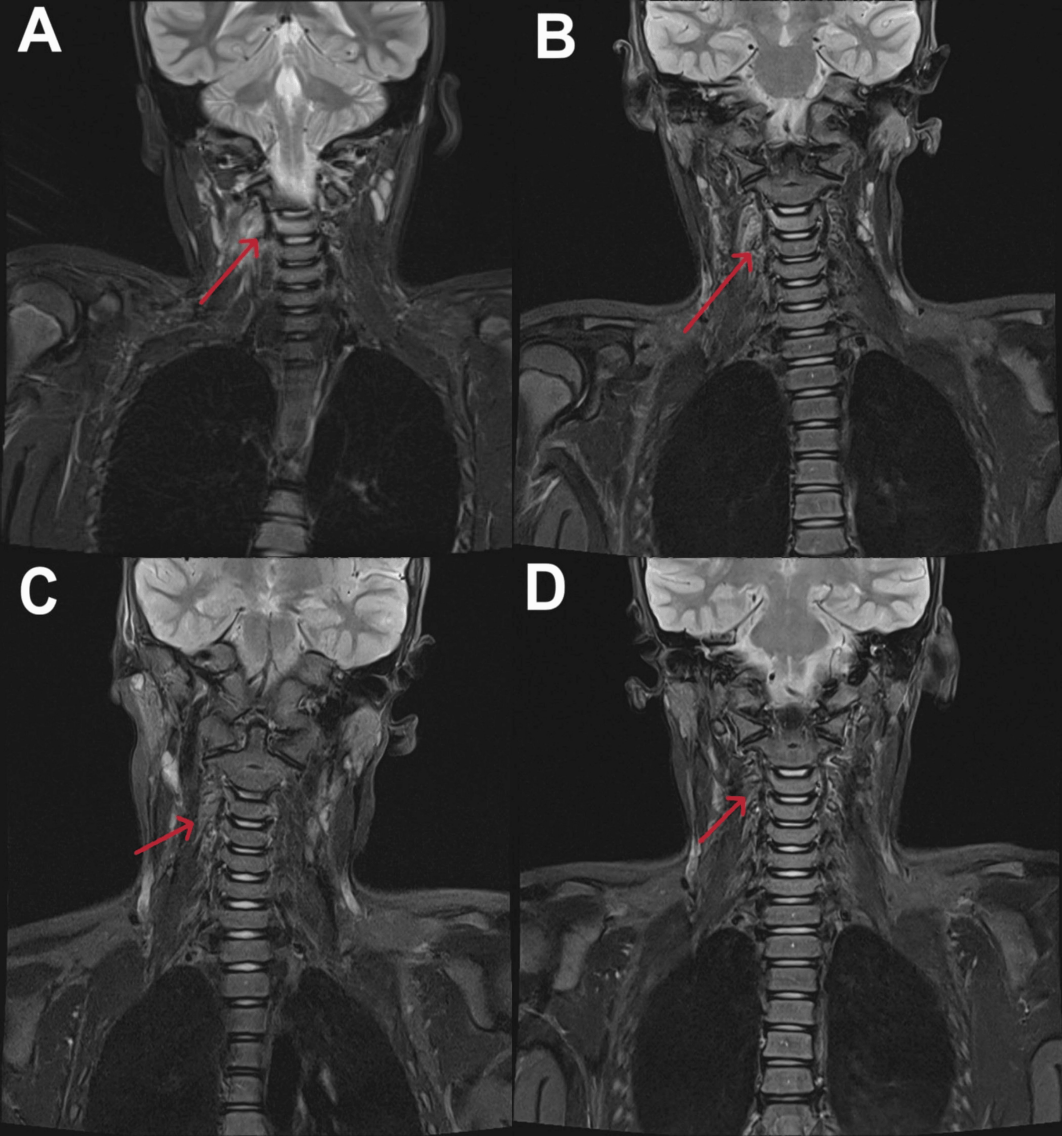
MRI images of the neck throughout the entire treatment course Image A - T2-weighted MRI of the neck showing hyperintensity involving the C3 vertebral body and right paravertebral soft tissues (arrow). No evidence of significant disc changes or adjacent collection was noted. Image B - T2-weighted MRI of the neck showing hyperintensity involving the C3 vertebral body, with mild, relatively decreased right-sided paravertebral inflammatory changes (arrow), without evidence of disease progression, necrotic changes, localised sizeable collection, or disc involvement. Image C - T2-weighted MRI of the neck showing a regressive course of the previously noted right paravertebral abnormal soft tissue signal (arrow). Image D - T2-weighted MRI of the neck showing no evidence of abnormal soft tissue signals or collections (arrow). No evidence of paravertebral soft tissue abnormal signal was noted. MRI, Magnetic Resonance Imaging

On day 6, a neck MRI showed reactive tonsils, adenoids, and retropharyngeal and axillary lymph nodes without necrosis. Prominent inflammatory changes were noted, with enhancement of the prevertebral and right-sided parapharyngeal soft tissues, as well as adjacent enhancing changes in the C3 and C4 vertebrae, without evidence of necrosis (Figure 2A). In view of these findings, ongoing fever and neck pain, treatment was escalated on day 7 to IV meropenem and vancomycin. A cervical spine MRI was performed to confirm the diagnosis, which showed high T2 and low T1 signal of the C3 vertebral body and the inferior facet of the C3 vertebral body and the superior facet of the C4 vertebral body on the right, indicating osteomyelitis.

Additional tests, including cytomegalovirus (CMV), human immunodeficiency virus (HIV), Epstein-Barr virus (EBV), random blood glucose, and tuberculosis (TB) (T-SPOT) tests, were negative, except for positive EBV IgG. Antistreptolysin O titre (ASOT) was 400 IU/mL (<200 IU/mL), and the initial erythrocyte sedimentation rate (ESR) was 77 mm/hr (0-10 mm/hr). Complement levels were within normal limits, with C3 at 1.36 g/L (normal: 0.9-1.8 g/L) and C4 at 0.37 g/L (normal: 0.1-0.4 g/L). On further investigation, a beta-D-glucan test was requested, which returned positive. This result was interpreted as a false positive, attributed to meropenem rather than an active fungal infection [5]. Lumbar puncture analysis was unremarkable, and urine culture was negative. Echocardiography showed no evidence of endocarditis. Nasal and inguinal swabs were obtained for methicillin-resistant *Staphylococcus aureus* (MRSA) screening and were negative. Examination revealed dental caries, which were initially suspected as a possible source of infection. 

**Figure 3:**
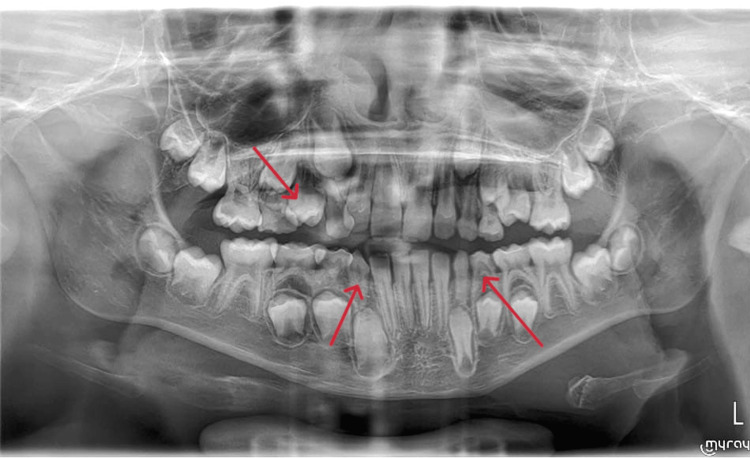
X-ray orthopantomogram showing dental caries with no deep-seated collection being identified (arrows)

An orthopantomogram X-ray was performed, demonstrating the extent of the dental caries, with no associated deep-seated collection (Figure 3). The Maxillofacial Team considered the caries unlikely to be the source of osteomyelitis and scheduled outpatient follow-up.

The patient continued to receive IV vancomycin and meropenem. On day 25, the patient exhibited some clinical improvement and a down-trending CRP. Therefore, the antibiotics were stepped down to IV ceftriaxone, oral metronidazole, and oral linezolid, followed by IV co-amoxiclav and oral metronidazole for a short duration.

However, he developed recurrent fever spikes and a rise in CRP from 17.7 mg/L to 213.3 mg/L within three days of antibiotic step-down. Due to clinical deterioration, the MDT recommended re-escalation from IV co-amoxiclav and oral metronidazole to IV vancomycin and meropenem, providing broader coverage, including resistant strains. These adjustments led to significant clinical and laboratory improvement, with eventual resolution of pain and fever. A repeat neck US scan showed no signs of abscess formation.

A repeat contrast-enhanced neck MRI demonstrated cervical vertebral findings, consistent with mild, relatively decreased right-sided parapharyngeal/prevertebral inflammatory changes, with no evidence of disease progression or localised sizeable collection. Additionally, unchanged adjacent enhancing C3 and C4 vertebral changes, with no necrotic changes, were noted (Figure 2B). Repeat blood and urine cultures showed no growth of organisms. One week later, a whole-body MRI was conducted to identify any other focus of infection, but no gross abnormalities were detected. As imaging studies revealed no evidence of a collection, the Neurosurgical Team concluded that no aspiration or other surgical procedure was warranted.

On day 36, IV vancomycin was replaced with IV teicoplanin to simplify therapeutic drug level monitoring, with once-daily dosing rather than multiple daily dosing. Subsequently, IV meropenem was switched to IV ertapenem.

**Figure 4:**
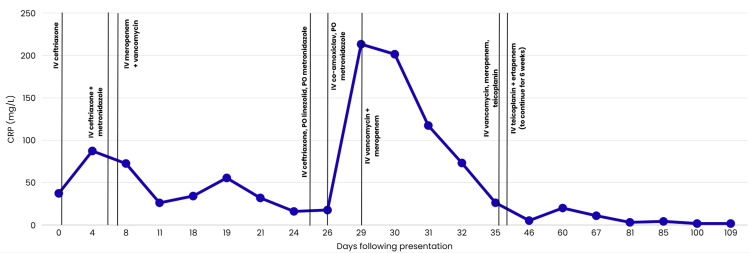
A summary of all antibiotic regimen changes, alongside CRP trends The figure depicts the CRP trend in relation to changes in antibiotic therapy. Initial escalation to intravenous (IV) vancomycin and meropenem on day 7 was associated with a good response; however, de-escalation on day 25 resulted in a rise in CRP, necessitating the re-escalation to IV vancomycin and meropenem. CRP, C-Reactive Protein

A summary image showing the antibiotic timeline, alongside the CRP trends, is shown in Figure 4.

On day 70, a repeat neck MRI with contrast showed a regressive course of the previously noted right perivertebral enhanced abnormal soft tissue signal. The right pedicle of C3 still showed increased short tau inversion recovery (STIR) signal enhancement (Figure 2C), which led the MDT to continue IV antibiotics for another six weeks.

The patient required four midline catheter insertions and multiple peripheral cannulations during treatment. The lines remained in situ for variable times, ranging from hours to weeks, which resulted in treatment interruptions. This, together with persistent MRI findings, warranted the MDT to extend IV antibiotic therapy. The total course lasted approximately 16 weeks, with several regimen adjustments to prevent potential complications. After six weeks of hospital admission, the patient was transferred to the paediatric virtual ward to continue IV antibiotic therapy at home under the Children’s Community Nursing Team.

A follow-up neck MRI with contrast, performed four months after admission, confirmed full resolution with no gross abnormalities (Figure 2D). The patient had a normal neurological examination throughout the entire treatment timeline. Follow-up at seven months confirmed full recovery, with no persistent neck pain.

## Discussion

Vertebral osteomyelitis is a rare condition in the paediatric population. The cervical region is less commonly affected when compared with the lumbar and thoracic regions [1,2].

In most paediatric cases, osteomyelitis results from the dissemination of bacteria via the blood from a remote focus. Less commonly, it results from trauma, and, rarely, from the direct spread of adjacent tissues, including otolaryngeal soft tissue infections [1,6]. Osteomyelitis in children is primarily caused by *Staphylococcus aureus* [3,7]. However, in younger children, *Kingella kingae* is a frequently recognised causative organism [8,9], while *Streptococcus* species are less common [7]. Other organisms, such as Gram-negative bacteria and *Bartonella henselae*, may be considered based on age, prevalence, clinical presentation, and underlying risk factors [2,10]. Fungi and *Mycobacterium tuberculosis* are rare causes [4], and polymicrobial infections involving anaerobes may occasionally occur secondary to contiguous spread from adjacent structures [6]. MRSA should always be considered due to its association with more complications [7].

The clinical presentation of cervical osteomyelitis in children is often subtle and nonspecific, which may lead to diagnostic delay [2,3]. Neck pain, fever, and torticollis are common presenting features [1]. In our case, the child initially presented with neck spasms, which later progressed to fever and cervical lymphadenitis, reflecting the variable, and sometimes misleading, clinical course of the disease. 

The diagnosis of vertebral osteomyelitis depends on a combination of clinical suspicion, inflammatory markers, and advanced imaging. Although inflammatory markers, including CRP and ESR, are non-specific, they are usually elevated in cases of osteomyelitis. Monitoring their trends is useful in following up the child's improvement but should be interpreted with the clinical course. A prolonged rise might be an indicator of a poorly treated source or suboptimal antibiotic coverage. CRP levels normalise earlier than ESR, which can remain elevated for a longer period [11,12]. 

Imaging is the mainstay in the diagnosis of osteomyelitis. MRI is considered the investigation modality of choice, as it outlines marrow oedema as well as intervertebral disc and soft tissue involvement [9,13]. When plain radiographs and US are compared, the former has poor sensitivity for acute disease and may even be normal in early disease [9]. The latter, although it has the ability to diagnose superficial and periosteal collections, has poor sensitivity for deep bony lesions [9,14]. CT scan imaging is of benefit in diagnosing retropharyngeal collection and cortical bony details, but it lacks sensitivity for early marrow changes [9,13]. When spinal osteomyelitis is suspected, MRI with contrast is the modality of choice to differentiate epidural or paravertebral masses from inflammatory lesions. Whole-body MRI should be considered if multifocal disease is a possibility [9]. This report illustrates the pivotal role and high sensitivity of MRI in the diagnosis of osteomyelitis.

In the described case, no definitive organisms were isolated, which is not uncommon in osteomyelitis. This demonstrates the need for empirical, broad-spectrum antibiotic therapy based on the most likely pathogens initially [7]. In certain cases, where blood cultures are negative or the source is uncertain, invasive sampling (such as aspiration or biopsy) may be necessary to identify the causative organism [3,12]. An MDT discussion, involving paediatric consultants, neurosurgeons, orthopaedic surgeons, an infectious disease specialist, and a microbiology consultant, was held. The decision not to perform a biopsy was made after weighing the risks and benefits, particularly in the absence of a definite collection.

While antibiotics remain the cornerstone of treatment for paediatric osteomyelitis, there have been multiple systematic reviews, randomised controlled trials, and observational studies comparing long IV therapy with short-duration IV therapy, followed by oral antibiotics, in the management of paediatric acute haematogenous osteomyelitis [15]. Evidence from clinical studies and guidelines supports early transition to oral antibiotics in cases of acute long bone osteomyelitis, guided by clinical picture, inflammatory markers, and causative organisms [7,16,17]. In some cases, including those involving neonates or young infants, MRSA infections, complicated presentations, or chronic osteomyelitis, a longer treatment course may be required [7,12]. Currently, there is limited evidence to support early transition to oral antibiotics in cases of acute vertebral osteomyelitis, and the clinical course is pivotal in planning management [7,12,17]. Surgical intervention may be required in certain cases of cervical osteomyelitis [18].

Our patient received a prolonged course of IV antibiotics due to recurrence of symptoms, variations in inflammatory markers, with CRP initially decreasing but subsequently rising after antibiotic step-down, persistent MRI changes, and the absence of an identified causative organism. The course was further complicated by interruptions in therapy due to difficulties with IV access. Some studies suggest that MRI findings may not immediately reflect clinical improvement in paediatric osteomyelitis. However, studies recommend correlating MRI results with the clinical course [19].

MDT involvement, including but not limited to paediatrics, orthopaedics, radiology, microbiology, neurosurgery, and infectious diseases teams if required, is an important aspect of the management of paediatric vertebral osteomyelitis and contributes to better outcomes [20]. Input from multiple teams was invaluable in our patient.

Although cervical osteomyelitis can be serious, the prognosis of paediatric vertebral osteomyelitis is often favourable, particularly if the condition is uncomplicated and managed promptly. However, long-term sequelae, such as neurological deficits, epidural abscess formation, spinal instability, and deformities, may occur in complicated cases [2,3].

## Conclusions

This case underscores that a high index of suspicion for cervical osteomyelitis is necessary in children with persistent neck pain. MRI is the imaging modality of choice, and management may require prolonged, tailored antibiotic therapy, guided by an MDT.

The major learning points from this case include the following: cervical vertebral osteomyelitis in children may present with subtle symptoms, leading to delayed diagnosis. Early MRI is essential when neck pain persists or worsens despite initial treatment, and management can be complicated by prolonged antibiotic therapy. MDT involvement is critical for timely diagnosis, individualised treatment planning, and monitoring recovery. Awareness of atypical presentations can improve clinical outcomes in rare paediatric spinal infections.
